# A randomized controlled trial examining a Tranquil sitting intervention compatible with Confucian values

**DOI:** 10.3389/fpsyg.2023.1118481

**Published:** 2023-07-03

**Authors:** Xiaochen Wen, Yaping Zhou, Yinan Li, Yue Lv, Siyi Han, Junshu Zhao

**Affiliations:** ^1^Department of Global and Area Studies, Pukyong National University, Busan, Republic of Korea; ^2^School of Philosophy and Sociology, Lanzhou University, Lanzhou, China; ^3^School of Management, Lanzhou University, Lanzhou, China; ^4^School of Chongqing Intellectual Property, Chongqing University of Technology, Chongqing, China; ^5^School of Philosophy and Religious Studies, Minzu University of China, Beijing, China; ^6^The Fourth People's Hospital, Ya'an, China

**Keywords:** sleep quality, tranquil sitting, Confucian method, self-cultivation, tranquillity and reverence

## Abstract

**Introduction:**

During the COVID-19 pandemic in China, the silent management (Lockdown) policy has caused severe sleep problems for university students. Long-term isolation may further deteriorate sleep quality, and it requires practical interventions. Today in mental and sleep health, interventions based on Buddhist, Taoist and Confucian ethics have been proven effective in reducing cognition and sleep disorders. However, such interventions also have limitations. They tend to focus on peace of mind or some technical means with the main direction of symptom improvement but neglect the mundane activities of daily life.

**Methods:**

We conducted an innovative tranquil sitting intervention program based on the Chinese Confucian value of the “tranquility and reverence” method, integrating various intervention techniques traditionally recognized as effective for achieving more lasting mental health and sleep quality. This study aims to assess the effectiveness and feasibility of a tranquil sitting intervention in improving sleep problems in isolated university students. Using a randomized control trial (RCT), the participants in the intervention program (*n* = 35) practiced the tranquil sitting intervention program for ten weeks. They had their PSQI scores measured at the pre-experimental, post-test, and 1-month follow-up time points and compared to the control group (*n* = 35).

**Results:**

The participants who received the tranquil sitting intervention had significantly better sleep quality than the control group, with moderate to large effect sizes in the middle and late stages. The instructor may challenge the intervention group at the beginning of the tranquil sitting technique. However, the improvement in sleep quality was significant after fully mastering the method.

**Discussion:**

The intervention program in this study emphasized the importance of “tranquility” and showed the same sleep improvement as in other traditional interventions. In conclusion, this intervention is a feasible and promising new approach to improving sleep quality among youths.

## Introduction

The worldwide COVID-19 pandemic has led to significant changes in public social lifestyles and has had a significant negative impact on public mental health and sleep quality (Targa et al., 2021). A meta-analysis showed that the total prevalence of sleep disorders in the global population was 35.7% and that the COVID-19 epidemic affected the sleep quality of approximately 40% of the general population and healthcare practitioners (Jahrami et al., 2021). As sleep disorders or problems associated with COVID-19 have become more prominent, researchers in various countries have paid increasing attention to it. They have begun to study sleep quality in different ages, occupations, regions, and status groups in depth (Qi et al., 2020; Duran and Erkin, 2021; Shillington et al., 2022).

Notably, poor sleep quality and psychological problems are risk factors for each other (Saadeh et al., 2021; Souza et al., 2021). Furthermore, there is growing evidence that lockdown policies related to the prevention of COVID-19 transmission show significant side effects on mental health and sleep issues, such as longer time to sleep, poorer sleep quality, and increased use of sleep medications (Alfonsi et al., 2021). In addition, mental health problems such as anxiety and stress caused by home quarantine also can exacerbate sleep problems (Xiao H. et al., 2020). Furthermore, it can even lead to insomnia and more severe mental health problems, perpetuating the mental and physical sequelae of sleep problems (Escobar-Córdoba et al., 2021; O'Regan et al., 2021).

University students are among the groups most widely affected by the COVID-19 lockdown. A study of sleep quality among university students during the lockdown showed that the prevalence of a Pittsburgh Sleep Quality Index (PSQI) > 5 increased from 80.6% before the lockdown to 82.7% afterwards. Furthermore, the mean PSQI score increased significantly (Khojasteh and Khadem-Rezaiyan, 2022). On the one hand, the quarantine policy restricted the daily lives of university students and reduced their physical activity, leading to sleep problems (Saguem et al., 2022).

Studies have shown that strict isolation negatively affects the mental health of university students and induces sleep disorders and depressive symptoms (Saadeh et al., 2021). Additionally, because mental health problems such as depression have a bidirectional relationship with sleep disorders, the youth’s mental health problems may also exacerbate their symptoms (Zou et al., 2020). Therefore, adequate sleep or psychological interventions are urgently needed to prevent further deterioration of sleep quality among university students (Ali et al., 2020).

Given the general population’s mental health and sleep problems, especially youth such as university students, caused by the lockdown policies, researchers have developed programmes or projects to mitigate the adverse effects. In the general population, COVID-19 patients are more likely to have serious psychological problems and sleep disorders due to stress and have received attention from many researchers. For example, progressive muscle relaxation (PMR) can significantly reduce anxiety and depression levels and improve patient sleep quality (Liu et al., 2020; Xiao C. X. et al., 2020). Furthermore, for the adolescent population, a peer education intervention (PEI) can improve anxiety, depression, and sleep quality (Ding and Yao, 2020).

Despite the good results of the above sleep interventions, two aspects deserve further discussion. First, regarding the intervention modality, the above interventions are still mainly in behavioural fields, where we could carry out other feasible programmes. Second, the intervention must be carried out based on ethical context, thus the varying cultural backgrounds of the subjects should raise attention. Furthermore, the intervention techniques should adjust to meet the participants’ cultural requirements.

In China, where Confucianism is a pervasive influence, a Confucian-based self-cultivation intervention approach has an advantageous cultural context and potential for successful implementation. Based on the knowledge above, this research uses the traditional Chinese Confucian “tranquillity and reverence” method as the foundation for creating a tranquil sitting intervention technique. In contrast to the mindfulness intervention model framework based on Buddhist ethics, this tranquil sitting intervention thoroughly adopts the Confucian ethic of “self-cultivation” and incorporates meditation techniques.

In order to maximize the potential of this inventive approach, the authors conducted a psychosocial sleep intervention programme that included social workers and counsellors to address the short-term and long-term sleeping difficulties experienced by university students due to the COVID-19 lockdown and to assess the efficacy of this tranquil sitting intervention technique.

## Background

### The ethical base of different interventions

The ideologies of Buddhism, Taoism, and Confucianism, known as the Three Teachings of East Asia, influence the cultural backgrounds, cognitive styles, and emotional expressions of East Asian populations at all times (Lin et al., 2021). In terms of psychological interventions, these influences are manifested in Mindfulness-Based Interventions (MBIs), Second-Generation Mindfulness-Based Interventions (SG-MBIs) and Taoist Cognitive Therapy (TCT). TCT has its roots in Taoist philosophy, while Buddhist teachings mainly characterise the framework of existing MBIs or SG-MBIs.

Buddhist positive meditation techniques and ethical guidelines are the sources of MBI. Initially, people apply MBI to treat depression, anxiety, chronic pain, and other psychopathologies (Wielgosz et al., 2019). It was later promoted to be applied to a broader range of psychological interventions and showed positive results in reducing stress, anxiety, depression and burnout (Liu et al., 1996; Lomas et al., 2019; Ding et al., 2020). However, the practitioners criticise the early MBIs for placing too much emphasis on positive meditation techniques at the expense of methodology, for example it compromises transparency and conflation of Buddhists with universal ideals (Brown, 2017). Learned from that, the new SG-MBIs have incorporated more Buddhist ethics (for instance, the four immeasurable meditation and the emptiness meditation). In addition, compared with the early MBIs, the new SG-MBIs emphasise cultivating a lifestyle (van Gordon et al., 2014, 2015). Relevant psychological intervention studies also have confirmed the effectiveness of SG-MBIs in reducing mental health problems, such as depressive symptoms and enhancing positive emotions (Lv et al., 2020).

Taoism significantly affected East Asian countries, particularly mainland China, where the TCT method was formulated. That amalgamates cognitive therapy with Taoist values and strives to help people adapt to their environment (Zhang and Yang, 1998). The Tao ethics, which include the principles of “Benefit not to harm,” “Nourish not to contend,” and “Be quiet and follow nature,” are employed mainly by TCT to bring about a change in personal values and facilitate psychological treatment (Chang et al., 2016; Chang et al., 2019; Wu and Lei, 2019). Other forms of Taoist practice, such as Taoist Tai Chi (TTC), Qigong and acupressure, have their roots in Taoist philosophy and have been proven to alleviate symptoms of psychological issues like anxiety, depression and sleep difficulty (Fogarty et al., 2016; Lai et al., 2017; Vera et al., 2019). TCT has been recognised for its intervention effects in the past 20 years. Studies involving sleep interventions have shown that both MBI and TCT can positively affect sleep disorders (Xia, 2018).

The recently emerging Mindfulness-Based Positive Psychology (MBPP) or BMAA interventions come from Confucian ethics (Teng and Lien, 2016; Zhou et al., 2021). The emphasis is on intervention with ethical clarification or integration of Confucian values, focusing more on traditional Confucian self-cultivation techniques. MBPP integrates Confucian values into SG-MBIs. And BMAA emphasises traditional Confucian self-cultivation methods.

### Tranquil sitting and meditation

As a physical practice, “meditation” is a form shared by Buddhism, Taoism and Confucianism (Meulenbeld, 2019). Taoism and Buddhism were the first to use the concept of “meditation,” so the standardised Confucian Tranquil sitting method developed during the Song and Ming dynasties was firmly related to Buddhism and Taoism. However, Confucian tranquil sitting differs markedly from Buddhism and Taoism meditation praxis in specific processes, goals and results. For example, the Confucian “tranquillity and reverence” method emphasises contemplation, nurturing, and physical concentration. The former requires that the consciousness level be quiet and awake, while the latter requires that the body level be solemn and neat so that the individual can achieve a state of unity (Yang, 2012). In the Confucian tranquil sitting method, the connotation transforms from “reverence” to “tranquillity,” the self-cultivation practice and the reasoning quasi-epistemological work combines, and the realisation of self-cultivation and moral principles produce a more concrete unification of the mind and body. Furthermore, although Confucianism also emphasises the elimination of personal “wild fancy” through tranquil sitting, it believes that the root cause of “wild fancy” is the interruption of the “harmony between man and nature” state. In contrast, tranquil sitting is the way back to the “harmony between man and nature” status. Only by doing this can the individual reflect on their “heavenly destiny” (the source of goodness) and eliminate the emotional changes and spiritual damage produced by “wild fancy.” In this sense, Confucianism aims to reconcile nature with humanity by emphasising personal tranquil sitting, and its emphasis on the ethical order is uncharacteristic of Buddhist and Taoist meditations.

Regarding the “tranquillity and reverence” method, Confucian tranquil sitting has yet to clarify detailed regulations on posture, breath, ashram (place), and time, the process of “nurturing” emphasised by Song and Ming scholars, which developed into the “tranquillity” and the “reverence” method (see Table 1). While in terms of tranquil sitting goals, it emphasises the state of mind and body during practice and implements it into daily life. In addition to the standard rule of tranquil sitting, the most prominent feature of Confucian tranquil sitting is the affirmation of the existence of the “secular world” and the “morality” so that the purpose of tranquil sitting is to make the “essence” manifest in the body and to put it into practice (Ma, 2012). Thus, the Confucian method of tranquil sitting differs from other meditation practices, for it has a primary intellectual orientation and the principle of behaviour (Yang, 2012).

**Table 1:** Confucian tranquil sitting method of “Tranquillity and Reverence.”

Category	Method	Mode	Purpose	Effect	Semantic mode
Undeveloped time	Static time	Self-restraint	Tranquil concealing the nature of the Divine Principle	Recognize the heavenly truth	Consciousness semantics
Developed time	Dynamic time	Evaluating	Moves and determines human desire in the slightest	Convergence of body and mind	Behavioural semantics

Son, Bycong-Ook, 2012, pp. 103–128.

### The necessity of tranquil sitting

An intervention must fit its cultural and ethical context to gain a satisfactory outcome. However, some of the values conveyed in the abovementioned interventions may conflict with Confucianism, one of the most influential traditions in the wider East Asian region, including mainland China. For example, certain aspects of Taoism, which originated in the Chinese mainland, have been refuted by Confucianism. Its well-known ethics of “out of the world,” expressed as the upekkha-equanimity meditation、metta-lovingkindness meditation、karuna-compassion meditation and mudita-empathetic joy meditation (Monteiro et al., 2019), is in contrast to the ethics of “self-prudence when alone” practised in mainland China (Lai, 2019), for the Confucianism is adherence to a more positive value, such as the practice of “inner sanctity,” which places greater emphasis on secular morality and social responsibility, as opposed to the “out of world” ideas.

In contrast to the transcendental teachings of Buddhism and Taoism, Confucianism emphasizes engaging in the secular world and reacting accordingly (Weber, 1959), and using tranquil sitting in order to preserve the ethical self-cultivation “Shendu (慎独)” (Han, 2023).MBPP and BMAA incorporate facets of Confucianism tradition and rituals. Although they merely scratched the surface of the traditional Confucian moral code, these techniques also inspired traditional Confucian interventions in Mainland China. However, these interventions still address the surface symptoms instead of treating the underlying cause.

It is crucial imports the “tranquillity and reverence” method parts from the MBPP method of “self-cultivation” and formulates the tranquil sitting intervention technique developed from the BMAA basis. In stark contrast to the MBPP and the BMAA, interventions that involve aspects of Confucianism are employed to mitigate cultural conflict. The synthesis of these two components produces an innovative approach based on the “tranquillity and reverence” method of tranquil sitting to bring about essential alterations in mental health and apply them to daily life. This novel intervention approach with an “inside out” perspective is a significant step forward from the traditional Chinese ethical psychological intervention developed since TCT, it focuses more on the inner workings of the individual than on external skills. Based on the “tranquillity and reverence” method, our study refers to its tranquil sitting form. It based on the established Buddhist and Taoist meditation intervention frameworks and experiences to create a set of tranquil sitting interventions applicable to the subjects to improve their sleep quality.

## Method

### Trial setting

We designed a randomized controlled experiment to test the Pittsburgh Sleep Quality Index (PSQI) changes of quarantined university students in the intervention and control groups. The experiment was conducted from September to November 2022 at a university undergoing “the silent management” (Lockdown) in Lanzhou city, Gansu Province of China. We mainly use the Tencent conference software for online communication, assisted by WeChat. Lanzhou is located in northwest China and is a critical node city of the Silk Road Economic Belt with 28 universities. From September to November 2022, there were severe outbreaks of COVID-19 in Lanzhou city. To reduce the risk of transmission, universities in the city adopted stricter lockdown measures and suspended all onsite teaching.

### Participants

The project participants were 88 enrolled university students from a university in Lanzhou city; 39 (44.3%) were male, 49 (55.7%) were female, aged 18 to 36 years (*M* = 23.78, SD = 3.32). We applied PASS 15 to calculate that an initial sample size of 44 participants (sample size estimation =15) in each group was employed at the beginning of this study, which was calculated based on *α* = 0.05, power = 0.9 (2-tailed test), intervention group pre-PSQI mean = 5.63, SD = 2.83, post-PSQI mean = 5.63, SD = 2.65 (according to Ding and Yao, 2020). When the programme finished, the intervention group’s power = 0.99 (T1 PSQI mean = 9.17, SD = 4.67, T3 PSQI mean = 5.06, SD = 2.49, *n* = 35). Following the outbreak of COVID-19 in Lanzhou city, these university students had to attend classes online and isolate themselves at school due to school closure.

Participants were recruited online mainly through a school-based student WeChat group, where they signed an informed consent form after being informed about the intervention programme and precautions. So, this is not a strictly randomized controlled experiment. Then we invited them to a WeChat group of intervention programme participants, social workers, and counsellors. Participants were selected based on the following criteria: (a) enrolled students (undergraduate, master’s, and doctoral students) who were at least 18 years old and experiencing campus closure; (b) not participating in a similar intervention programme; (c) entirely volunteering for the intervention programme, (d) in good health and without serious mental health problems and (e) self-reported as no religion.

The research subjects had to take three assessments: the pre-experiment (T1), post-test (T2), and one-month follow-up test (T3) (hereafter referred to as T1, T2, and T3). 70 (79.55%) individuals from the intervention programme finished the data collection at all three points. The 88 enrolled and isolated university students were randomly distributed into two groups: the intervention group received instructions on the tranquil sitting technique, and the control group received simple interactive body moves.

**Figure 1:**
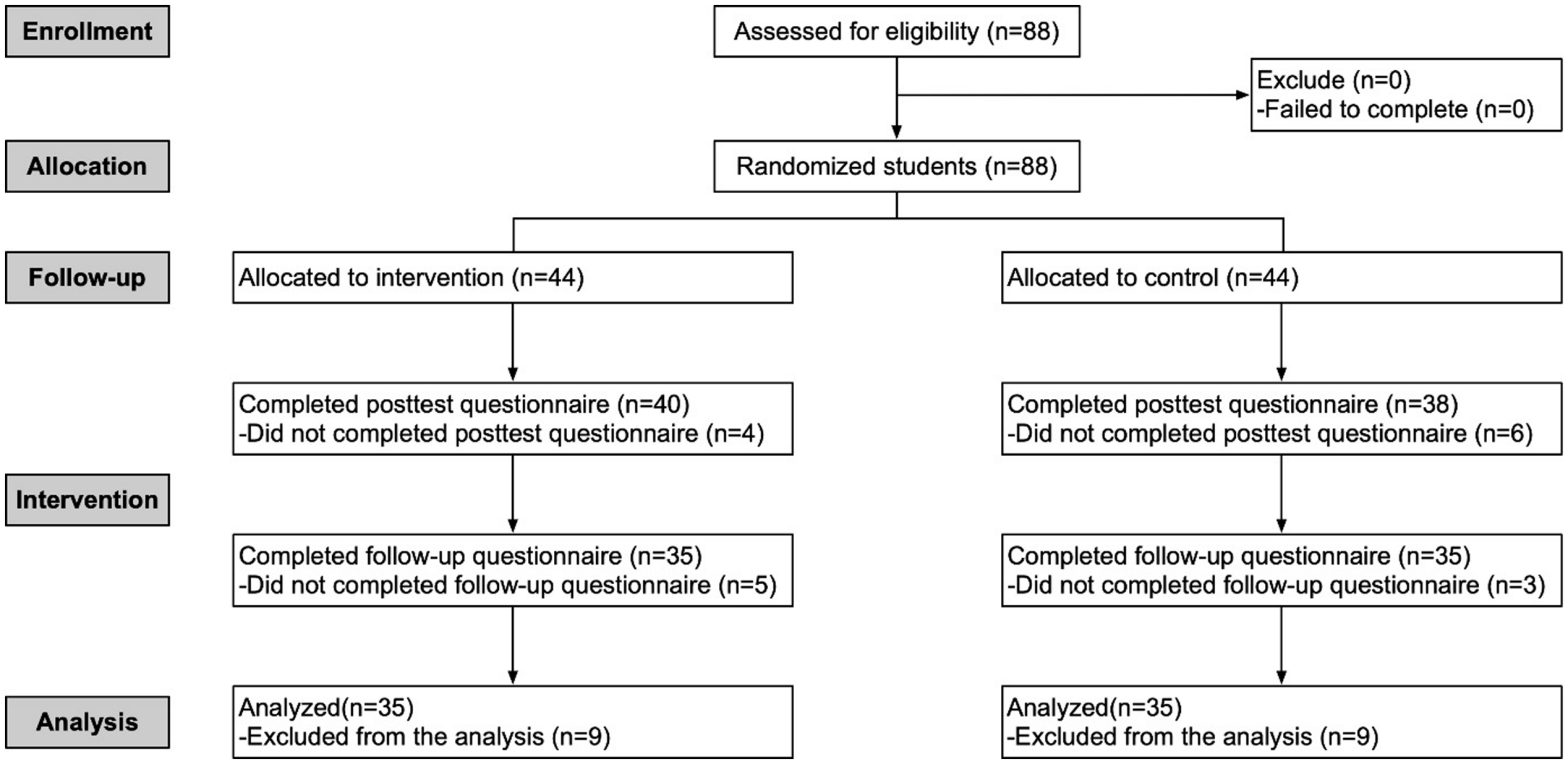
Flow of participants in the study.

Nine participants in the intervention group and nine in the control group failed to complete data collection during the intervention period, so we excluded these participants from the analysis. Therefore, the experiment retained data from 70 participants ultimately. Include 30 (42.9%) males and 40 (57.1%) females, aged 18 to 32 years (*M* = 23.56, SD = 3.13) (see Table 2 for demographic information on the sample and Figure 1 for the flow chart).

**Table 2:** The differences of participants at the pre-experimental (T1).

Variables		All subjects (*n* = 70)	Intervention (*n* = 35)	Control (*n* = 35)	*p*
Age (mean ± SD)		23.56 ± 3.13	24.00 ± 3.23	23.11 ± 3.01	0.215*
Gender (%)	Male	30 (42.9%)	14 (40.0%)	16 (45.7%)	0.629
	Female	40 (57.1%)	21 (60.0%)	19 (54.3%)	
Degree (%)	Bachelor	22 (31.4%)	8 (22.9%)	14 (40.0%)	0.302**
	Master	34 (48.6%)	19 (54.2%)	15 (42.9%)	
	PhD	14 (20.0%)	8 (22.9%)	6 (17.1%)	
Quarantine (%)	1–2 weeks	4 (5.8%)	3 (8.5%)	1 (2.9%)	0.184**
	3-4 weeks	15 (21.4%)	8 (22.9%)	7 (20.0%)	
	5–6 weeks	22 (31.4%)	7 (20.0%)	15 (42.9%)	
	7 weeks +	29 (41.4%)	17 (48.6%)	12 (32.2%)	
Birthplace (%)	Big city	17 (24.3%)	7 (20.0%)	10 (28.6%)	0.457**
	Medium city	25 (35.7%)	11 (31.4%)	14 (40.0%)	
	Town	7 (10.0%)	5 (14.3%)	2 (5.7%)	
	Countryside	21 (30.0%)	12 (34.3%)	9 (25.7%)	
PSQI (mean ± SD)		8.94 ± 4.33	9.17 ± 4.67	8.71 ± 4.03	0.063*

* Wilcoxon signed-rank test.

** Chi-squared.

Participants did not differ in gender between the intervention and control groups (*p* = 0.629) or in mean age (*p* = 0.215). There were no differences between groups on PSQI score (*p* = 0.063), degree(*p* = 0.302), quarantine(*p* = 0.184) and birthplace(*p* = 0.457) (see Table 2). There was no significant difference in PSQI between the intervention and control groups at T1 (see Table 2, T1 Wilcoxon *Z* = 1.86, *p* = 0.063). The Human Subjects Ethics Committee of the School of Philosophy and Social Sciences approved the protocol at Lanzhou University.

### Measures and analysis

We assessed participants’ sleep quality in the intervention programme using the Pittsburgh Sleep Quality Index (PSQI) (Buysse et al., 1989). The PSQI was developed based on the analysis of various scales for assessing sleep quality and is composed of seven components(19 items, range of subscale scores, 0–3): sleep quality, sleep latency (i.e., time needed to fall asleep), sleep duration (i.e., number of hours of actual sleep per night), sleep efficiency (i.e., total sleep time divided by time in bed, converted to a score 0–3), sleep disturbance (i.e., waking up in the middle of the night), sleep medication and sleep dysfunction (i.e., having difficulty staying awake during the day). The sum of these component scores yields a global score (range of scores, 0–21). The PSQI has shown good internal reliability when applied to studies related to sleep quality in China (Wang J. et al., 2022; Wang L. et al., 2022). In the present study, the Cronbach’s α values for T1, T2 and T3 were 0.844, 0.852 and 0.882, respectively, indicating that the overall internal consistency of the PSQI in the present study was good.

SPSS 20.0 was used to analyse the programme data. The skewness and kurtosis test indicates that the intervention and control groups follow the approximately normal distribution at T1, T2 and T3. Therefore, we can utilise the repeated measures analysis. The Shapiro–Wilk test suggested that the PSQI and its entry data were skewed at T1, T2, and T3, prompting the paired-samples Wilcoxon test to be used to determine the change in PSQI scores at the three points stated above (Rice and Harris, 2005). We used Cohen’s *r* to calculate the effect size, with values of 0.1 representing a minor effect size, 0.3 signifying a moderate effect size, and 0.5 or higher indicating a substantial effect size (Coolican, 2018, p. 395).

### Design of the intervention

When the universities enforce strict lockdown management, they entail students staying on campus, minimizing contact with each other and even remaining in dormitories if necessary. University students frequently suffer from anxiety, depression, and insomnia due to this regulation (Saraswathi et al., 2020). The correlation between psychological problems and sleep disturbances is mutual, and China has already seen some positive sleep intervention results based on Confucian culture (Chu and Carew, 1990). The tranquil sitting intervention technique proposed in this study is close to MBI, SG-MBI and TCT in form, advocates Confucian ethics of “tranquillity and reverence” in self-cultivation and pursues a “tranquillity” state to alleviate psychological problems or sleep disorders.

Even with the university lockdown, students must manage multiple duties in their lives and studies, making it hard to achieve peace of mind. Therefore, the authors crafted this intervention promotes tranquillity and encourages individuals to incorporate self-cultivation to manage their daily lives. In the formation of psychological tranquillity, the primary focus is on integrating the physical, emotional, and mental states.

The objective of evaluating morality is to contemplate and respond to daily life with it. Upon receiving guidance from the social worker tutors, the participants quickly learned to align the body, regulate their breath, and focus their cognition after becoming accustomed to the tranquil sitting. Self-cultivation helped strengthen their mental fortitude and capacity to cope (Hwang and Chang, 2009). With the help of the social worker instructors, once the subjects experienced the tranquil sitting state, they mastered the exercises of “body alignment,” “breath regulation,” and the “cognitive focus” (Teng and Lien, 2016). Their mental resilience and coping ability improved through the tranquillity of self-cultivation. Thus, their sleep quality was enhanced from the inside outward.

The programme integrates both group and individual interventions. Group intervention can furnish mutual support (Supiano and Luptak, 2014), so it was primarily conducted in group mode. However, some participants may find it challenging to express their inner thoughts and needs in the groups. Thus, each social worker instructor maintained one-on-one contact with about eight subjects *via* SMS or WeChat. Moreover, every participant had the opportunity to receive individualized online assistance besides the group intervention. This initiative helps build trust and informs the director about each participant’s mental health and sleep status.

The whole programme consists of 14 topics and lasts for 10 weeks, with two sessions of about 50 min each week. The intervention includes the following six sections: 1. Explore the cultural roots of Confucianism. 2. An overview of the Confucian tranquil sitting ritual and the underlying ethics. r3. A primer on Confucian methodologies of “tranquillity and reverence” tranquil sitting. 4. Practice Confucian “body alignment,” “breath regulation,” and “cognition focus” techniques. 5. Practice engaging in Confucian tranquil sitting with the “body, breath, cognition” method, contemplation and response. 6. Review of the intervention programme. Table 3 shows the specific intervention content.

**Table 3:** Overview of Confucian tranquil sitting practice.

Period	Theme	Point
Week1	Overview of tranquil sitting	Introduction to the history, types, effects, and contents of tranquil sitting
	Ethics of meditation	Buddhist, Taoist and Confucian meditation ethics, differences & relationships, etc.
Week2	Confucian tranquil sitting	Confucian method of tranquil sitting representation
	Ethics of the Confucian tranquil sitting	Ethics of Tranquil sitting
Week3	“Tranquillity”	Method of “tranquillity”
	“Reverence”	Method of “reverence”
Weeks4-5	“Body alignment”	Practice the “body alignment” (posture)
	“Breath regulation”	Practice the “breath regulation” (breath)
	Cognitive focus	Practice the “cognitive focus” (cognition)
Weeks6-9	Self-cultivation	Integrating the body, emotions and feelings into the “self-cultivation” tranquil sitting
	Moral inspection	Integrating the contemplation and response into the moral inspection tranquil sitting
	“Tranquillity and reverence”	Deepen knowledge of “tranquillity and reverence”
	Confucian tranquil sitting comprehensive practice	Tranquil sitting comprehensive practice
Week10	Exercise summary and experience reflection	Postintervention support, ongoing practice and review of the intervention

The fourth and fifth sections were the focus of the intervention, which lasted 6 weeks. The intervention group participated in two 50-min tranquil sitting sessions per week. The control group interacted simultaneously and in the same format without the instructor guiding them to tranquil sitting. The intervention was carried out strictly according to the plan developed by the social worker and the directors, with fun and interactive sessions during and after the intervention to ensure its effectiveness and the participants’ attendance.

## Results

The result of Mauchly’s sphericity test shows that Machly W = 0.903 while *p* = 0.033. Therefore, we use the correction in Greenhouse-Geisse. Table 4 shows the primary outcome measures. On our primary outcome PSQI score, the repeated measures analysis showed a significant within-subject effect for time [*F* = 22.343, *p* < 0.001, *η*^2^= 0.247] but no effect for group [*F* = 2.630, *p* = 0.109, *η*^2^ = 0.037]. The time*group effect for the PSQI score was statistically significant [*F* = 23.912, p < 0.001, *η*^2^ = 0.260].

**Table 4:** Outcome data and results of the repeated measures analysis.

	T1	T2	T3	ANOVA of repeated measuring		
	(mean ± SD)	(mean ± SD)	(mean ± SD)	*F*	*p*	*η* ^2^
Intervention	9.17 ± 4.67	8.14 ± 4.15	5.06 ± 2.49			
Control	8.71 ± 4.03	9.09 ± 3.91	8.89 ± 4.45			
Time				22.243	0.000	0.247
Group				2.630	0.109	0.037
Time*group				23.912	0.000	0.260

**Figure 2:**
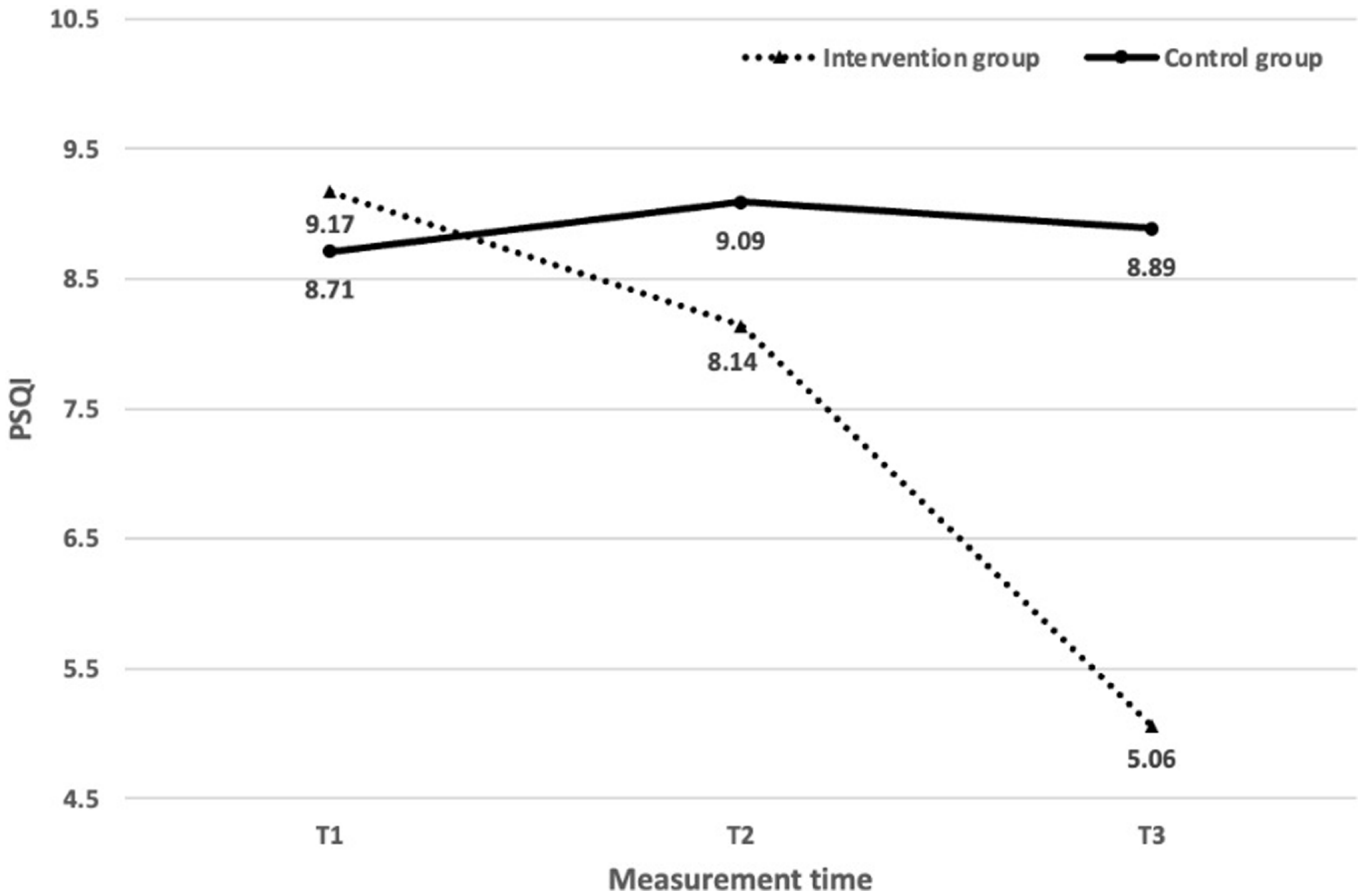
PSQI mean during the three phases of the study.

We subsequently performed the simple effect test. The two groups showed no significant differences at T1[*F* = 0.192, *p* = 0.662, *η*^2^ = 0.003] and T2[*F* = 0.956, *p* = 332, *η*^2^ = 0.014], however a significant difference was observed at T3[*F* = 19.745, p < 0.001, *η*^2^ = 0.225]. Furthermore, the intervention group demonstrated significant variations at various time intervals [*F* = 38.879, *p* < 0.001, *η*^2^ = 0.537], whereas the control group showed no such significance [*F* = 0.487, *p* = 0.616, *η*^2^ = 0.014]. Multiple comparisons revealed that the PSQI scores of the intervention group decreased significantly at T2 and T3 when compared to T1[T2-T1: SMD = −1.029, 95%CI (−1.947, −0.110); T3-T2: SMD = -3.086, 95%CI (−4.300, −1.872)]. Conversely, the control group did not experience any significant shifts in their PSQI scores [T2-T1: SMD = 0.371, 95%CI (−0.547, 1.290); T3-T2: SMD = -0.200, 95%CI (−1.414, 1.014)]. Figure 2 illustrates a significant drop in the PSQI scores of the intervention group.

Test results of the PSQI scores evidence a significant difference between T2 and T3 compared to T1. The individuals in the intervention group had a moderate decrease in their PSQI scores at T2 (*Z* = 2.98, *p* = 0.003, Cohen’s *r* = 0.47). The measurements of T3 showed a more significant decrease in PSQI scores (*Z* = 4.89, *p* = 0.000, Cohen’s *r* = 0.77), suggesting a significant advancement in sleep quality among these students from T1 to T3. The PSQI scores of the intervention group remained unchanged in most items, apart from Sleep Latency, Sleep Duration, Sleep Disturbance, Sleep Medication, Sleep Dysfunction from T1 to T2, and Sleep Efficiency from T2 to T3. The scores of Sleep Latency, Sleep Duration, Sleep Disturbance, Sleep Medication, Sleep Dysfunction, and Sleep Efficiency remained relatively constant from T2 to T3, whereas the other scores had significant alterations and considerable effect sizes (*Z* [2.11, 4.74]; Cohen’s *r* [0.33, 0.75]), indicating that while some PSQI scores had no significant alterations from T1 to T2, they did change significantly from T2 to T3, as Table 5 describes.

**Table 5:** Changes in intervention group from pre-experimental (T1) to one-month follow-up (T3).

	T3-T1			T2-T1			T3-T2		
	*z*	*p*	*r*	*z*	*p*	*r*	*z*	*p*	*r*
PSQI	4.89	0.000	0.77	2.98	0.003	0.47	4.64	0.000	0.73
Sleep Quality	4.51	0.000	0.71	2.71	0.007	0.43	4.74	0.000	0.75
Sleep Latency	4.09	0.000	0.65	1.37	0.175	0.22	3.65	0.000	0.58
Sleep Duration	2.11	0.035	0.33	0.00	1.000	0.00	2.33	0.020	0.37
Sleep Efficiency	2.49	0.013	0.39	2.32	0.020	0.37	0.91	0.366	0.14
Sleep Disturbance	3.74	0.000	0.59	1.34	0.180	0.21	3.32	0.001	0.52
Sleep Medication	2.53	0.011	0.40	0.58	0.564	0.09	2.33	0.020	0.37
Sleep Dysfunction	3.41	0.001	0.54	0.00	1.000	0.00	3.88	0.000	0.61

The PSQI scores of the control group at T2 and T3 showed no significant difference compared to T1 (T2-T1: *Z* = 0.91, *p* = 0.365; T3-T1: *Z* = 0.51, *p* = 0.610), and there was also no marked change at T3 when compared to T2 (T3-T2: *Z* = 0.64, *p* = 0.521). Additionally, the PSQI scores of the control group did not exhibit any significant alterations except for sleep quality from T2 to T3 and sleep dysfunction from T1 to T3, as Table 6 reveals. In summary, The PSQI and its respective entry scores of the intervention group showed a significant improvement compared to the control group.

**Table 6:** Changes in control group from pre-experimental (T1) to one-month follow-up (T3).

	T3-T1			T2-T1			T3-T2		
	*z*	*p*	*r*	*z*	*p*	*r*	*z*	*p*	*r*
PSQI	0.51	0.610	0.08	0.91	0.365	0.14	0.64	0.521	0.10
Sleep Quality	0.36	0.719	0.06	1.61	0.108	0.25	1.97	0.049	0.31
Sleep Latency	1.21	0.225	0.19	0.84	0.400	0.13	0.27	0.790	0.04
Sleep Duration	1.63	0.102	0.26	0.23	0.819	0.04	1.70	0.090	0.27
Sleep Efficiency	0.00	1.000	0.00	0.60	0.552	0.09	0.53	0.597	0.08
Sleep Disturbance	0.68	0.499	0.11	0.73	0.467	0.12	0.00	1.000	0.00
Sleep Medication	0.63	0.527	0.10	1.61	0.108	0.25	0.79	0.430	0.12
Sleep Dysfunction	2.06	0.040	0.31	1.81	0.071	0.29	0.34	0.735	0.05

## Discussion

The purpose of this research is to observe how participation in Confucian “tranquillity and reverence” tranquil sitting could improve the sleep quality of university students in COVID-19 quarantine. Our findings indicated that the 10-week intervention programme led to a gradual enhancement of sleep quality among the intervention group from T1 to T2 to T3, relative to the control group. Despite the intervention group experiencing a significant decrease in PSQI scores, the mean PSQI score at T3 was 5.06, which was still higher than the threshold value of 5, suggesting that the university students need to continue improving their sleep quality even after the intervention programme. On the other hand, the control group, which did not receive the tranquil sitting instruction, had a mean PSQI score of 8.89, significantly higher than the threshold of 5. In addition, the intervention group’s average scores of PSQI and most of its components were significantly lower than those of the control group. This result suggests that the tranquil sitting intervention could be a promising way to enhance the sleep quality of quarantined university students.

Furthermore, a mean PSQI score of 8.94 was discovered for all the subjects when measured at T1, suggesting that the lockdown policy has detrimentally impacted the sleeping quality of university students, similar to the findings in other relevant studies (Bi et al., 2022; Wang D. et al., 2022). It is noteworthy that the results of this research surpass the rate (80%) of poor sleep quality (PSQI>5) among university students at the start of the COVID-19 pandemic (Zhang et al., 2020; Zhou et al., 2020), Indicates that sleep quality problems among Chinese university students have become more severe over time. The worsening mental health issues among university students possibly relate to the COVID-19 pandemic isolation, reduced physical activity and higher academic stress (Yin et al., 2022). The fact that university students are not getting enough sleep demonstrates the need for immediate action, and our research validates the necessity and effect of this tranquil sitting intervention.

This research reveals that the PSQI scores of the intervention group significantly dropped, and the effect sizes are significant when measured at each point from T1 to T3. We found that the tranquil sitting program, rooted in Chinese Confucian “tranquillity and reverence” ethics, made a marked difference in the sleep quality of university students during the quarantine period. The impact of the intervention varied between stages of the programme. The intervention group showed a notable improvement in PSQI scores from T2 to T3, while the change from T1 to T2 was moderate. Most PSQI components displayed no significant variation from T1 to T2, while all the items excluding sleep efficiency exhibited a significant alteration from T2 to T3. The causes below may be associated with the outcomes above. Almost all the subjects’ sleep problems were severe before joining the programme. The critical objective of phase one was to foster trust between the participants, the social work tutors and the programme director.

After the programme initiation, the intervention group members gradually became accustomed to the tranquil sitting technique based on more abstract and intricate Confucianism ethics. Between T2 and T3, the intervention group acquired the knowledge of the Confucian tranquil sitting technique, the “tranquillity and reverence” ethics, and the “body, breath, and cognition” coordination method. They engaged in integrated tranquil sitting practices during the programme. The knowledge they have gained regarding intervention ethics and their experience with the tranquil sitting technique may be the essential factors that lead to the significant decrease in PSQI scores and the scores of each item from T2 to T3.

Moreover, psychological or sleep interventions enhance adolescent sleep quality (Ding and Yao, 2020). Our research results align with investigations that utilized other interventions with different populations during the COVID-19 pandemic (Liu et al., 2020; Xiao C. X. et al., 2020). MBIs and SG-MBIs have a Buddhist ethical basis, and TCT has a Taoist one. They all include a sleep component in their interventions. In the former case, research demonstrated that MBI effectively improved sleep quality before the COVID-19 pandemic but not during the pandemic (Lim et al., 2021), perhaps indicating participants’ urgency for the intervention. In the latter case, the acupressure intervention based on the Taoist ethic positively impacted sleep quality (Lai et al., 2017). Similar to the above studies, the Tranquil sitting intervention of this study achieved positive sleep intervention results in the more severe Lockdown conditions, suggesting that this intervention method has a broad application potential.

## Limitations, strengths, and further research

This research determined that the tranquil sitting technique was efficacious in enhancing the sleep quality of university students. However, it would be wise to approach the conclusions of this paper with caution. First, the paper could not adhere to a strict random sampling protocol due to operational constraints. Consequently, the sample could potentially be biased. Second, this research investigated the relationship between sleep quality and mental health. However, it did not assess the mental health of the individuals involved, which could weaken the impact of the tranquil sitting technique on psychological well-being. Thirdly, as this is the initial phase of the tranquil sitting research, we did not analyse the participants’ demographic attributes and sleeping habits. We could consider incorporating these variables into our future research design. Finally, the interaction between instructors and subjects is mainly through the internet. The lack of understanding of the ethics of tranquil sitting due to this limitation may have hindered the efficacy of the intervention.

Despite the limitations of this study, it still makes specific contributions. On the one hand, in addition to intervention methods such as MBI, BMAA and TCT, this study presents the tranquil sitting intervention based on traditional Chinese Confucian ethics for the first time. On the other hand, it constitutes an intervention based on the “tranquillity and reverence” ethics as the core, “body, breath and cognition” coordination as the mean, and “connotation and inspection” as the methodology, with contemplation and response in the secular world. This innovative technique emphasises the ethical “secularity” of the method in everyday practice but also caters to the psychological and developmental needs of university students to a certain extent and achieves significant sleep intervention results, which are ground-breaking.

On the other hand, the authors also test the feasibility of traditional Confucianism as the intervention ethics through the tranquil sitting technique. It can spark innovative approaches for mental health and sleep interventions in East Asian societies and can serve as a blueprint for further exploration. Researchers in this field could explore novel approaches to addressing mental health issues in various groups and make this technique more efficient and reliable.

## Conclusion

This study reveals that a tranquil sitting intervention utilizing the Chinese Confucian ethics of “tranquillity and reverence” can effectively enhance the sleep quality of university students in quarantine. Thus, it supports the implementation of other mainstream sleep or psychological intervention approaches. Consequently, we advocate utilizing the tranquil sitting derived from Chinese Confucian principles of “tranquillity and reverence” in East Asian societies, as it has been proven successful in the form of an Internet-based intervention programme. It could also be applied onsite. Furthermore, as this intervention approach is the first of its kind, we recommend expanding the ethical framework to encompass a broader range of populations, thereby creating more practical and hybrid strategies.

## Data availability statement

The datasets presented in this article are not readily available because the data is required not to share by the supporting fund. Requests to access the datasets should be directed to ypzhou@lzu.edu.cn.

## Ethics statement

The studies involving human participants were reviewed and approved by the Human Subjects Ethics Committee of the School of Philosophy and Social Sciences at Lanzhou University. The patients/participants provided their written informed consent to participate in this study.

## Author contributions

XW was responsible for data collection and writing the thesis. YZ provided the overall idea for the research. YiL completed the experimental design and data analysis. YuL and JZ are registered social workers responsible for the clinical intervention. SH sorted out and translated knowledge of Confucian ethics. All authors contributed to the article and approved the submitted version.

## Funding

This research is sponsored by the National Social Science Grant of China: 22BSH068.

## Conflict of interest

The authors declare that the research was conducted in the absence of any commercial or financial relationships that could be construed as a potential conflict of interest.

## Publisher’s note

All claims expressed in this article are solely those of the authors and do not necessarily represent those of their affiliated organizations, or those of the publisher, the editors and the reviewers. Any product that may be evaluated in this article, or claim that may be made by its manufacturer, is not guaranteed or endorsed by the publisher.

## Supplementary material

The Supplementary material for this article can be found online at: https://www.frontiersin.org/articles/10.3389/fpsyg.2023.1118481/full#supplementary-material

## References

[ref1] Alfonsi V. Gorgoni M. Scarpelli S. Zivi P. Sdoia S. Mari E.. (2021). COVID-19 lockdown and poor sleep quality: not the whole story. J. Sleep Res. 30:e13368. doi: 10.1111/jsr.13368, PMID: 33955081 PMC8236908

[ref2] Ali K. Mufti U. Mufti A. (2020). Impact of COVID-19 lockdown on sleep quality in students: a cross sectional study. Ann. Int. Med. Dental Res. 6:1. doi: 10.21276/aimdr.2020.6.6.PH1

[ref3] Bi C. Lin H. Zhang J. Zhao Z. (2022). Association between sleep quality and depression symptoms in Chinese college students during the COVID-19 lockdown period. Children 9:1237. doi: 10.3390/children9081237, PMID: 36010127 PMC9406988

[ref4] Brown C. G. (2017). Ethics, Transparency, and Diversity in Mindfulness Programs. In: eds Monteiro, L., Compson, J., and Musten, F. Practitioner’s Guide to Ethics and Mindfulness-Based Interventions (pp. 45–85). Mindfulness in Behavioral Health. Springer, Cham. doi: 10.1007/978-3-319-64924-5_3

[ref5] Buysse D. J. Reynolds C. F. 3rd Monk T. H. Berman S. R. Kupfer D. J. (1989). The Pittsburgh Sleep Quality Index: a new instrument for psychiatric practice and research. Psychiatry Res. 28, 193–213. doi: 10.1016/0165-1781(89)90047-4, PMID: 2748771

[ref6] Chang D. F. Hung T. Ng N. Ling A. Chen T. Cao Y.. (2016). Taoist cognitive therapy: treatment of generalized anxiety disorder in a Chinese immigrant woman. Asian Am. J. Psychol. 7, 205–216. doi: 10.1037/aap0000052

[ref7] Chang D. F. Miao I. Y. Cao Y. (2019). Returning to the source: the indigenization of Chinese Taoist cognitive psychotherapy in the United States [追本溯源:中国道家认知疗法在美国的本土化]. Chinese. J. Clin. Psychol. [中国临床心理学杂志]. (03), 644–646+643. doi: 10.16128/j.cnki.1005-3611.2019.03.044

[ref8] Chu F.-Y. K. Carew R. (1990). Confucianism: its relevance to social work with Chinese people. Aust. Soc. Work. 43, 3–9. doi: 10.1080/03124079008550085

[ref9] Coolican H. (2018). Research methods and statistics in psychology (7th). London: Routledge

[ref10] Ding Y. Wang L. Chen J. Zhao J. Guo W. (2020). Chinese Taoist cognitive therapy for symptoms of depression and anxiety in adults in China: a systematic review and Meta-analysis. Front. Psychol. 11:769. doi: 10.3389/fpsyg.2020.00769, PMID: 32390917 PMC7192096

[ref11] Ding X. Yao J. (2020). Peer Education Intervention on Adolescents’ Anxiety, Depression, And Sleep Disorder During the Covid-19 Pandemic. Psychiatr Danub. 32, 527–535. doi: 10.24869/Psyd.2020.527, PMID: 33370763

[ref12] Duran S. Erkin Ö. (2021). Psychologic distress and sleep quality among adults in Turkey during the COVID-19 pandemic. Prog. Neuro-Psychopharmacol. Biol. Psychiatry 107:110254. doi: 10.1016/j.pnpbp.2021.110254, PMID: 33485962 PMC7825837

[ref13] Escobar-Córdoba F. Ramírez-Ortiz J. Fontecha-Hernández J. (2021). Effects of social isolation on sleep during the COVID-19 pandemic. Sleep. Science 14, 86–93. doi: 10.5935/1984-0063.20200097, PMID: 34917279 PMC8663737

[ref14] Fogarty J. N. Murphy K. J. McFarlane B. Montero-Odasso M. Wells J. Troyer A. K.. (2016). Taoist tai chi® and memory intervention for individuals with mild cognitive impairment. J. Aging Phys. Act. 24, 169–180. doi: 10.1123/japa.2014-0062, PMID: 25838271

[ref15] Han S. (2023). “Tranquillity” and “reverence” in the theory of method: meaning and mechanism. Working paper.

[ref16] Hwang K. K. Chang J. (2009). Self-cultivation: culturally sensitive psychotherapies in Confucian societies. Couns. Psychol. 37, 1010–1032. doi: 10.1177/0011000009339976

[ref17] Jahrami H. BaHammam A. S. Bragazzi N. L. Saif Z. Faris M. Vitiello M. V. (2021). Sleep problems during the COVID-19 pandemic by population: a systematic review and meta-analysis. J. Clin. Sleep Med. 17, 299–313. doi: 10.5664/jcsm.8930, PMID: 33108269 PMC7853219

[ref18] Khojasteh M. R. Khadem-Rezaiyan M. (2022). Evaluation of sleep quality before and during COVID-19 quarantine and its relationship with family social support: a web-based survey among university students. Iran. J. Psychiatry Behav. Sci. 16:e123447. doi: 10.5812/ijpbs-123447

[ref19] Lai Q. (2019). On the three approaches to Confucian cultivation - from the three meanings of caution, fear, and prudence in the Zhongyong [论儒家修身工夫的三种进路——从《中庸》戒、惧、慎独三义说起]. Philos. Res. [哲学研究]. 11, 47–53.

[ref20] Lai F. C. Chen I. H. Chen P. J. Chen I. J. Chien H. W. Yuan C. F. (2017). Acupressure, sleep, and quality of life in institutionalized older adults: a randomized controlled trial. J. Am. Geriatr. Soc. 65, e103–e108. doi: 10.1111/jgs.14729, PMID: 28152177

[ref21] Lim J. Leow Z. Ong J. Pang L. S. Lim E. (2021). Effects of web-based group mindfulness training on stress and sleep quality in singapore During the COVID-19 Pandemic: Retrospective Equivalence Analysis. JMIR Ment Health. 8:e21757. doi: 10.2196/21757, PMID: 33482627 PMC7962857

[ref22] Lin Y. Y. Swanson D. P. Rogge R. D. (2021). The Three Teachings of East Asia (TTEA) inventory: developing and validating a measure of the interrelated ideologies of Confucianism, Buddhism, and Taoism. Front. Psychol. 12:626122. doi: 10.3389/fpsyg.2021.626122, PMID: 33732190 PMC7956942

[ref23] Liu K. Chen Y. Wu D. Lin R. Wang Z. Pan L. (2020). Effects of progressive muscle relaxation on anxiety and sleep quality in patients with COVID-19. Complement. Ther. Clin. Pract. 39:101132. doi: 10.1016/j.ctcp.2020.101132, PMID: 32379667 PMC7102525

[ref24] Liu X. Tang M. Hu L. Wang A. Wu H. Zhao G.. (1996). Reliability and validity of Pittsburgh sleep quality index [匹兹堡睡眠质量指数的信度和效度研究]. Chin. J. Psychiatry [中华精神科杂志]. 29, 103–107.

[ref25] Lomas T. Medina J. C. Ivtzan I. Rupprecht S. Eiroa-Orosa F. J. (2019). Mindfulness-based interventions in the workplace: an inclusive systematic review and meta-analysis of their impact upon wellbeing. J. Posit. Psychol. 14, 625–640. doi: 10.1080/17439760.2018.1519588

[ref26] Lv J. Liu Q. Zeng X. Oei T. P. S. Liu Y. Xu K.. (2020). The effect of four immeasurables meditations on depressive symptoms: a systematic review and meta-analysis. Clin. Psychol. Rev. 76:101814. doi: 10.1016/j.cpr.2020.101814, PMID: 31945711

[ref27] Ma Y. Y. (2012). “Confucian views on sit-in and the rise of the idea of the unity of three religions in Song and Ming dynasties [宋明时期儒学对静坐的看法以及三教合一思想的兴起]” in The tradition of meditation sitting in East Asia [东亚的静坐传统]. eds. Yang R. Ma Y. Y. Halvor E. (Taipei: Publishing Center, National Taiwan University), 63–102.

[ref28] Meulenbeld M. (2019). Confucianism, Daoism, Buddhism, and Chinese Popular Religion. In Oxford Research Encyclopedia of Asian History. doi: 10.1093/acrefore/9780190277727.013.126

[ref29] Monteiro L. M. Musten F. Leth-Steensen C. (2019). Effect of mindfulness on value incongruence: a pilot study. Mindfulness 10, 1031–1043. doi: 10.1007/s12671-018-1044-7

[ref30] O'Regan D. Jackson M. L. Young A. H. Rosenzweig I. (2021). Understanding the impact of the COVID-19 pandemic, lockdowns and social isolation on sleep quality. Nat. Sci. Sleep 13, 2053–2064. doi: 10.2147/NSS.S266240, PMID: 34795545 PMC8593898

[ref31] Qi J. Xu J. Li B. Z. Huang J. S. Yang Y. Zhang Z. T.. (2020). The evaluation of sleep disturbances for Chinese frontline medical workers under the outbreak of COVID-19. Sleep Med. 72, 1–4. doi: 10.1016/j.sleep.2020.05.023, PMID: 32502844 PMC7244440

[ref32] Rice M. E. Harris G. T. (2005). Comparing effect sizes in follow-up studies: ROC Area, Cohen's d, and r. Law Hum. Behav. 29, 615–620. doi: 10.1007/s10979-005-6832-7, PMID: 16254746

[ref33] Saadeh H. Saadeh M. Almobaideen W. Al Refaei A. Shewaikani N. Al Fayez R. Q.. (2021). Effect of COVID-19 quarantine on the sleep quality and the depressive symptom levels of university students in Jordan during the spring of 2020. Front. Psych. 12:605676. doi: 10.3389/fpsyt.2021.605676, PMID: 33664681 PMC7920987

[ref34] Saguem B. N. Nakhli J. Romdhane I. Nasr S. B. (2022). Predictors of sleep quality in medical students during COVID-19 confinement. L'Encephale 48, 3–12. doi: 10.1016/j.encep.2021.03.001, PMID: 33863513 PMC8828364

[ref35] Saraswathi I. Saikarthik J. Senthil Kumar K. Madhan Srinivasan K. Ardhanaari M. Gunapriya R. (2020). Impact of COVID-19 outbreak on the mental health status of undergraduate medical students in a COVID-19 treating medical college: a prospective longitudinal study. PeerJ 8:e10164. doi: 10.7717/peerj.10164, PMID: 33088628 PMC7571415

[ref36] Shillington K. J. Vanderloo L. M. Burke S. M. Ng V. Tucker P. Irwin J. D. (2022). Not so sweet dreams: adults' quantity, quality, and disruptions of sleep during the initial stages of the COVID-19 pandemic. Sleep Med. 91, 189–195. doi: 10.1016/j.sleep.2021.02.028, PMID: 33685852 PMC9017869

[ref01] Son Bycong-Ook. (2012). “The Tranquility Method of Korean Confucianism [韩国儒学之静坐法]” in The tradition of meditation sitting in East Asia [东亚的静坐传统]. eds. R. Yang, Y. Y. Ma and E. Halvor (Taipei: Publishing Center, National Taiwan University), 103–128.

[ref37] Souza L. F. F. Paineiras-Domingos L. L. Melo-Oliveira M. E. S. Pessanha-Freitas J. Moreira-Marconi E. Lacerda A. C. R.. (2021). The impact of COVID-19 pandemic in the quality of sleep by Pittsburgh Sleep Quality Index: a systematic review. Cien. Saude Colet. 26, 1457–1466. doi: 10.1590/1413-81232021264.45952020, PMID: 33886773

[ref02] Supiano K. P. Luptak M. (2014). Complicated grief in older adults: A randomized controlled trial of complicated grief group therapy. The Gerontologist, 54, 840–856., PMID: 23887932 10.1093/geront/gnt076

[ref38] Targa A. D. S. Benítez I. D. Moncusí-Moix A. Arguimbau M. de Batlle J. Dalmases M.. (2021). Decrease in sleep quality during COVID-19 outbreak. Sleep Breath. 25, 1055–1061. doi: 10.1007/s11325-020-02202-1, PMID: 32989674 PMC7521946

[ref39] Teng S. C. Lien Y. W. (2016). What Confucius practiced is good for your mind: examining the effect of a contemplative practice in Confucian tradition on executive functions. Conscious. Cogn. 42, 204–215. doi: 10.1016/j.concog.2016.03.01627038245, PMID: 27038245

[ref40] Van Gordon W. Shonin E. Griffiths M. D. (2015). Towards a second generation of mindfulness-based interventions. Aust. N. Z. J. Psychiatry 49, 591–592. doi: 10.1177/0004867415577437, PMID: 25801660

[ref41] Van Gordon W. Shonin E. Sumich A. Sundin E. C. Griffiths M. D. (2014). Meditation awareness training (MAT) for psychological well-being in a sub-clinical sample of university students: a controlled pilot study. Mindfulness 5, 381–391. doi: 10.1007/s12671-012-0191-5

[ref42] Vera F. M. Manzaneque J. M. Rodríguez F. M. Vadillo M. Navajas F. Heiniger A. I.. (2019). Assessment of hormonal parameters and psychological well-being in healthy subjects after a Taoist qigong program: an exploratory study. Scand. J. Psychol. 60, 43–49. doi: 10.1111/sjop.12501, PMID: 30428134

[ref43] Wang J. Wang Y. Shen K. Li W. (2022). “Cognitive behavioral therapy in mainland China” in Cognitive behavioral therapy in a global context. eds. Terjesen M. D. Doyle K. A. (Cham: Springer)

[ref44] Wang L. Wu Y.-X. Lin Y.-Q. Wu Y. X. Lin Y. Q. Wang L.. (2022). Reliability and validity of the Pittsburgh sleep quality index among frontline COVID-19 health care workers using classical test theory and item response theory. J. Clin. Sleep Med. 18, 541–551. doi: 10.5664/jcsm.9658, PMID: 34534069 PMC8805004

[ref45] Wang D. Zhao J. Zhai S. Huang S. Yang Z. Pan Y.. (2022). Longitudinal trajectories of insomnia symptoms among college students during the COVID-19 lockdown in China. J. Psychosom. Res. 157:110795. doi: 10.1016/j.jpsychores.2022.110795, PMID: 35364373 PMC9386300

[ref46] Weber M. (1959): The religion of China. Confucianism and Taoism. Translated by Hans H. Gerth 2nd. Glencoe, IL: The Free Press.

[ref47] Wielgosz J. Goldberg S. B. Kral T. R. A. Dunne J. D. Davidson R. J. (2019). Mindfulness meditation and psychopathology. Annu. Rev. Clin. Psychol. 15, 285–316. doi: 10.1146/annurev-clinpsy-021815-093423, PMID: 30525995 PMC6597263

[ref48] Wu R. Lei M. (2019). A Review of Chinese Daoistic Cognitive Therapy [中国道家认知疗法的应用研究现状与展望]. Psychology: Techniques and Application [心理技术与应用]. 7, 693–700. doi: 10.16842/j.cnki.issn2095-5588.2019.11.007

[ref49] Xia W. (2018). Taoist Cognitive Therapy in Mainland China. Philos Pract: Journal of the APPA. 13, 2142–2152.

[ref50] Xiao C. X. Lin Y. J. Lin R. Q. Liu A. N. Zhong G. Q. Lan C. F. (2020). Effects of progressive muscle relaxation training on negative emotions and sleep quality in COVID-19 patients: A clinical observational study. Medicine, 99:e23185. doi: 10.1097/MD.0000000000023185, PMID: 33217826 PMC7676563

[ref51] Xiao H. Zhang Y. Kong D. Li S. Yang N. (2020). Social capital and sleep quality in individuals who Self-Isolated for 14 Days During the Coronavirus disease 2019 (COVID-19) Outbreak in January 2020 in China. Medical science monitor: international medical journal of experimental and clinical research. 26:e923921. doi: 10.12659/MSM.923921, PMID: 32194290 PMC7111105

[ref52] Yang R. (2012). “Reverence and tranquillity [主敬与主静]” in The tradition of meditation sitting in East Asia [东亚的静坐传统]. eds. Yang R. Ma Y. Y. Halvor E. (Taipei: Publishing Center, National Taiwan University), 129–160.

[ref53] Yin F. Chen C. Song S. Chen Z. Jiao Z. Yan Z.. (2022). Factors affecting university students’ sleep quality during the normalisation of COVID-19 epidemic prevention and control in China: a cross-sectional study. Sustainability 14:10646. doi: 10.3390/su141710646

[ref54] Zhang Y. Yang D. (1998). Chinese daoistic cognitive therapy: an introduction of the ABCDE techniques [中国道家认知疗法——ABCDE技术简介]. J. Chin, Psychol. Health 12, 61–63.

[ref55] Zhang Y. Young D. Lee S. Zhang H. Xiao Z. Hao W.. (2002). Chinese taoist cognitive psychotherapy in the treatment of generalized anxiety disorder in contemporary China. Transcult. Psychiatry 39, 115–129. doi: 10.1177/136346150203900105

[ref56] Zhang Y. Zhang H. Ma X. Di Q. (2020). Mental health problems during the COVID-19 pandemics and the mitigation effects of exercise: a longitudinal study of college students in China. Int. J. Environ. Res. Public Health 17:3722. doi: 10.3390/ijerph17103722, PMID: 32466163 PMC7277113

[ref57] Zhou S. J. Wang L. L. Yang R. Yang X. J. Zhang L. G. Guo Z. C.. (2020). Sleep problems among Chinese adolescents and young adults during the coronavirus-2019 pandemic. Sleep Med. 74, 39–47. doi: 10.1016/j.sleep.2020.06.001, PMID: 32836185 PMC7274988

[ref58] Zhou J. Zheng Y. Zeng X. Jiang M. Oei T. P. S. (2021). A randomized controlled trial examining a second-generation mindfulness-based intervention that is compatible with Confucian values: mindfulness-based positive psychology. Mindfulness 12, 1412–1423. doi: 10.1007/s12671-021-01610-y

[ref59] Zou P. Wang X. Sun L. Liu K. Hou G. Yang W.. (2020). Poorer sleep quality correlated with mental health problems in college students: a longitudinal observational study among 686 males. J. Psychosom. Res. 136:110177. doi: 10.1016/j.jpsychores.2020.110177, PMID: 32623194

