# Supplementary material for: A randomized controlled trial examining a Tranquil sitting intervention compatible with Confucian values
Source: Front Psychol. 2023 Jul 3;14:1118481. doi: 10.3389/fpsyg.2023.1118481 (PMC10352322; doi:10.3389/fpsyg.2023.1118481)
Supplement: Supplementary file 1 [file Data_Sheet_1.pdf]

Appendix 1: supplementary material

Buddhist positive meditation techniques and ethical guidelines are the sources of MBI. Initially, people apply MBI to treat depression, anxiety, chronic pain, and other psychopathologies (Wielgosz et al., 2019). It was later promoted to be applied to a broader range of psychological interventions and showed positive results in reducing stress, anxiety, depression and burnout (Lomas et al., 2019). However, the practitioners criticise the early MBIs for placing too much emphasis on positive meditation techniques at the expense of ethics. Learned from that, the new SG-MBIs have incorporated more Buddhist ethics (for instance, the four immeasurable meditation and the emptiness meditation). In addition, compared with the early MBIs, the new SG-MBIs emphasise cultivating a lifestyle (van Gordon et al., 2015).

The widely-using MBI has matured in continuous improvement. Relevant psychological intervention studies also have confirmed the effectiveness of SG-MBIs in reducing mental health problems, such as depressive symptoms and enhancing positive emotions (Lv et al., 2020). In addition, SG-MBIs began to focus on the role of intervention ethics on intervention effectiveness, which laid the foundation for developing MBPP. Thus, MBPP is the product of rethinking SG-MBIs. In particular, some of the values conveyed in SG-MBIs may conflict with Confucianism, one of the most influential traditions in the wider East Asian region, including mainland China.

To overcome the criticism of traditional Confucianism in applying SG-MBIs in East Asia, SG-MBI gradually replaces MBPP with a new connotation. Its psychological intervention, with the addition of Confucian elements such as interpersonal relationships and virtue ethics, significantly reduced the anxiety and stress levels of the subjects, making it an applicable and promising SG-MBI for promoting the mental health of Chinese people (Zhou et al., 2021).

Compared to MBPP, BMAA is more characteristics regarding its methodology and ethics. BMAA was developed based on Ya Le dance**1**, which originated from the music and dance used in ancient Chinese rituals to worship the heavens and was later transformed into part of the traditional Confucian "Six Arts" to cultivate the noble qualities. Moreover, the ultimate goal of achieving the "unity of heaven and man" and the long-term practice of BMAA can lead to greater mental peace and a sense of well-being and satisfaction (Teng & Lien, 2016).

Studies have indicated that TCT has been effective in reducing symptoms of a generalised anxiety disorder (GAD) in the long term (Zhang et al., 2002), as well as having a positive effect on depression (Ding et al., 2020). TCT's positive outcomes have led to its adoption in

---

[1] Ya Le dance, 雅乐舞

the US and European countries, making it a viable option for addressing mental health issues. Furthermore, it denoted the completion of the systemisation of TCT. Research demonstrated that subjects' symptoms of psychological issues, like stress and anxiety, were notably ameliorated after receiving systematic TCT interventions (Chang et al., 2016).

Studies have indicated that TCT has been effective in reducing symptoms of a generalised anxiety disorder (GAD) in the long term (Zhang et al., 2002), as well as having a positive effect on depression (Ding et al., 2020). TCT's positive outcomes have led to its adoption in the US and European countries, making it a viable option for addressing mental health issues. Furthermore, it denoted the completion of the systemisation of TCT. Research demonstrated that subjects' symptoms of psychological issues, like stress and anxiety, were notably ameliorated after receiving systematic TCT interventions (Chang et al., 2016).

"Tranquillity and Reverence" includes the connotation of "tranquil sitting" as a practice and is a common and essential foundation (Lin, Swanson & Rogge, 2021). While "connotation of the undeveloped mind" means "recognising the heavenly truth", specifically, "recognising the undeveloped causes of happiness, anger, sadness, and joy", and ultimately "conceives the nature of the sanctity Principle". This kind of quiet time work represents the semantics of consciousness in Confucianism's method theory (Han, 2023). To cope with the above dangers and overcome the defects of the Reverent connotation method, Song and Ming scholars proposed the "Tranquillity and Reverence" method, which is a connotation method that runs through the undeveloped time (static status) and the developed time (dynamic status).

In the self-cultivation of " tranquillity and reverence", it is necessary to sit in a tranquil status to nurture sympathetic consciousness when it is not yet mature and to examine the "already developed" stage. It also is necessary to collect the body and mind during the "developed" stage of examination to facilitate a complementary relationship with the "perfection of reason". Therefore, the "tranquillity and reverence" method put "inspection" into the ethical practice. At this time, the tranquil sitting practice can bring about the effect of convergence of body and mind and awakening consciousness.

Therefore, it relates to the next stage of self-cultivation, the "perfection of reasoning". In this process, there is bound to be a connection with daily behaviour, so the "reverent method" can be dynamic work. The mode of tranquil sitting represents the semantics of behaviour in Confucianism's method theory. In this sense, "tranquillity and reverence" are both Confucian practices leading to "inner sanctity " and can be unified as "reverence and reasoning", which have both dynamic and static meanings. Several theorists represent the standard of tranquil sitting following Confucian ethics (see Table 2).

Table 2: Confucian normalized Tranquil sitting example

Name	Method	Methodological foundation	Points
Zhu Xi 朱熹	Method of breathing	Positive thinking	Body regulation and curing diseases
Wang Ji 王畿	Breath regulation	The four phases of rest in Tiantai Buddhism	Tranquil sitting is an initiation to the mind with breath regulation
Yan Jun 颜钧	Seven-day retreat method	Taoist “internal circulation” technique	Rest for seven days after Tranquil sitting to the limit
Yuan Huang 袁黄	Tips for tranquil sitting	“Four Immeasurable Meditations” in Tiantai Buddhism	Connecting tranquil sitting to Confucianism
Gao Panlong 高攀龙	Tranquil sitting for seven days	Seven days to recover in The Book of Changes	Tranquil sitting for seven days to focus the cognition

The abovementioned interventions prioritise resolving psychological issues and sleep disturbances rather than concentrating on secular daily life. The mindfulness technique is the most mainstream psychological sleep intervention, which has evolved into meditation-based ethics and is continually refined by exploring new ethical foundations (Van Gordon et al., 2014, 2015; Monteiro et al., 2019). East Asian cultures have produced their own methods of psychological sleep therapy, such as Taoist cognitive therapy (TCT) and body-mind axial awareness (BMAA), that consider the region's unique cultural perspectives. The ethical principles behind mindfulness intervention and TCT originate from Buddhism and Taoism. They utilise similar intervention tactics of sitting concurrently, with TCT relying on forgetfulness and BMAA using meditation (Zhang et al., 2002; Ding et al., 2020; Teng & Lien, 2016). TCT has facilitated the implementation of Confucian-based interventions in mainland China that align with the traditional Chinese notion of "method"(Doris et al., 2019; Chang et al., 2016).
